# Role of Monocyte/Macrophages during HIV/SIV Infection in Adult and Pediatric Acquired Immune Deficiency Syndrome

**DOI:** 10.3389/fimmu.2017.01693

**Published:** 2017-12-05

**Authors:** Kristen M. Merino, Carolina Allers, Elizabeth S. Didier, Marcelo J. Kuroda

**Affiliations:** ^1^Division of Immunology, Tulane National Primate Research Center, Covington LA, United States; ^2^Division of Microbiology, Tulane National Primate Research Center, Covington LA, United States

**Keywords:** HIV, SIV, macrophages, monocytes, pediatrics, Acquired Immune Deficiency Syndrome

## Abstract

Monocytes/macrophages are a diverse group of cells that act as first responders in innate immunity and then as mediators for adaptive immunity to help clear infections. In performing these functions, however, the macrophage inflammatory responses can also contribute to pathogenesis. Various monocyte and tissue macrophage subsets have been associated with inflammatory disorders and tissue pathogeneses such as occur during HIV infection. Non-human primate research of simian immunodeficiency virus (SIV) has been invaluable in better understanding the pathogenesis of HIV infection. The question of HIV/SIV-infected macrophages serving as a viral reservoir has become significant for achieving a cure. In the rhesus macaque model, SIV-infected macrophages have been shown to promote pathogenesis in several tissues resulting in cardiovascular, metabolic, and neurological diseases. Results from human studies illustrated that alveolar macrophages could be an important HIV reservoir and humanized myeloid-only mice supported productive HIV infection and viral persistence in macrophages during ART treatment. Depletion of CD4+ T cells is considered the primary cause for terminal progression, but it was reported that increasing monocyte turnover was a significantly better predictor in SIV-infected adult macaques. Notably, pediatric cases of HIV/SIV exhibit faster and more severe disease progression than adults, yet neonates have fewer target T cells and generally lack the hallmark CD4+ T cell depletion typical of adult infections. Current data show that the baseline blood monocyte turnover rate was significantly higher in neonatal macaques compared to adults and this remained high with disease progression. In this review, we discuss recent data exploring the contribution of monocytes and macrophages to HIV/SIV infection and progression. Furthermore, we highlight the need to further investigate their role in pediatric cases of infection.

## Introduction

A cure for HIV infection continues to be elusive, despite the use of antiretroviral therapy (ART). Studies using non-human primate (NHPs) models of simian immunodeficiency virus (SIV) have been invaluable in better understanding the pathogenesis of this infection. Declining numbers of CD4+ T cells during HIV/SIV infections have long been attributed as the primary cause of immune-deficiency, progression to acquired immune deficiency syndrome (AIDS), and sites of virus reservoirs established during ART. While it is likely that various other immune cells influence disease progression and viral seeding, the purpose of this review is to focus on the contribution of monocytes and macrophages to HIV/SIV infection. Although in adult macaques, monocyte levels in blood remain constant during infection, the monocyte turnover rate increases during the onset of simian AIDS (SAIDS) and reflects damage to short-lived tissue macrophages. Interestingly, physiological monocyte turnover rate in neonates (<1 month old) is higher than in adults and appears to be associated with the more rapid disease progression that occurs in pediatric HIV/SIV infections. As infants became older (<1 year old), they exhibited a biphasic pattern of monocyte turnover and disease progression, with some animals presenting with rapid disease such as seen in neonates, while others progressed similarly as adult macaques. We theorize that the magnitude of infected macrophages influences disease outcome, and susceptibility of macrophages to infection is impacted by age. Our data suggest shorter-lived macrophages are more easily destroyed by virus while longer-lived macrophages appear more resistant to destruction and may contribute to the viral reservoir. This review discusses the possible contributions of macrophages to HIV/SIV infection and pathogenesis. It is important to consider that macrophages also may need to be targeted to achieve cure.

## Macrophages in Health and Disease

The immune system is essential for maintaining health and combatting infectious disease (1). Innate immune responses typically act immediately to produce inflammation and are performed directly by immune surveillance by effector cells such as neutrophils, natural killer (NK) cells, and macrophages. The innate immune cells work en masse to detect and clear pathogens and this is thought to limit the initial infection and thus buys time for adaptive immune responses to mount. Adaptive immune responses generally require weeks-to-months to develop and rely heavily on antigen-presenting cells (e.g., dendritic cells, macrophages, B cells) and T cells to clear infection and establish immunologic memory.

Macrophages are a diverse group of cells that play important roles in both innate and adaptive immune responses. During innate immune responses, macrophages sense and promote inflammation and recruit effector cells for clearance of pathogens. In adaptive immune responses, they function in antigen presentation and secretion of immune modulators for recruitment of lymphocytes (1). In addition, macrophages are imperative for tissue modeling during fetal development, maintaining homeostasis, and wound healing (2–4). Conversely, dysfunctional macrophages and unregulated inflammation are devastating to homeostasis and may instead contribute to pathogenesis in specific disease settings, such as pulmonary tissue injury during asthma (5), islet cell destruction in diabetes (6), and neurological damage, lymphoma and cardiovascular disease during human immunodeficiency virus (HIV) infection [reviewed in Ref. (7)].

**Figure 1:**
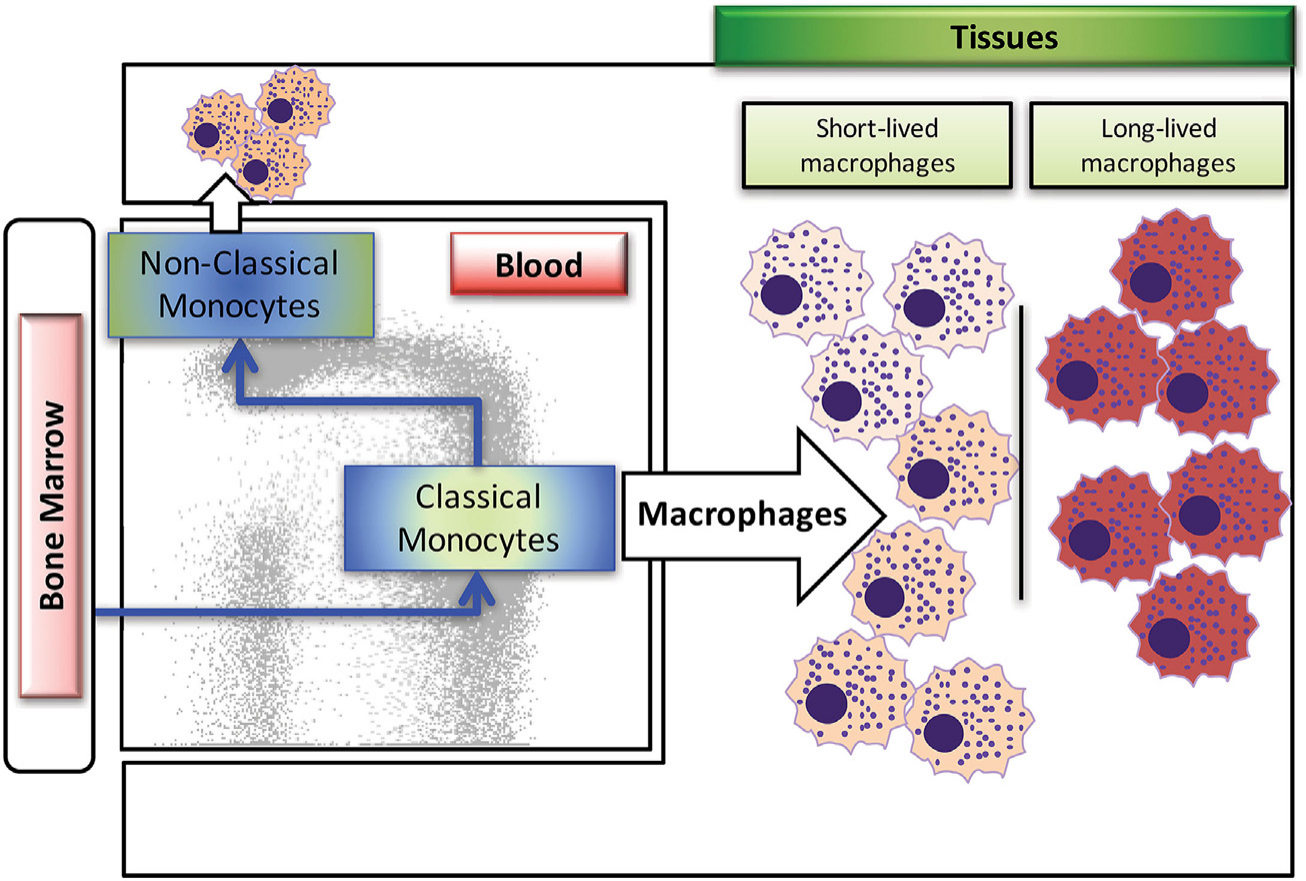
Summary of blood monocyte and dendritic cells (DC) differentiation in rhesus macaques. Myeloid precursor cells in the bone marrow migrate into blood and give rise to CD14^+^CD16^−^ classical monocytes. A large proportion of these classical monocytes rapidly disappear from the circulation to become tissue macrophages. A fraction of the classical monocytes differentiate into CD14^+^CD16^+^ intermediate monocytes and then into CD14^−^CD16^+^ non-classical monocytes in the circulation. The populations of mDCs, pDCs, and perhaps other unidentified DCs appear to differentiate from various DC precursors directly in the bone marrow prior entering the blood circulation. Figure modified from Ref. (8). Copyright 2015. The American Association of Immunologists, Inc.

Macrophages are derived from multiple sources during development, with earliest generation from the yolk sac during embryogenesis, and later generation from the fetal liver and bone marrow. In human and rhesus macaque adults, replenishment of tissue macrophages lost from death due to infection or injury occurs mainly from monocytes generated by hematopoietic stem cells in the bone marrow, which then circulate through the blood stream, deposit into the tissues, and differentiate into various types of macrophages (8–10). Macrophages in the mouse model have been shown to self-renew rather than being replaced with emigrating monocytes from the periphery (11–13). Based on published reports in murine models, tissue-resident macrophages can originate from two sources, with the self-renewing subset derived from the fetal liver during embryonic development and from the bone marrow after birth [reviewed in Ref. (10, 14)]. Studies have yet to identify self-renewing macrophages in primates, with possible exception of the brain (15). As summarized in Figure 1, there exist at least two types of macrophages in the tissues; the short-lived macrophages that are continuously replenished by circulating classical monocytes arising from the bone marrow and the long-lived macrophages. These two types of macrophages appear to differ in their phenotypes and functions, so it is critical to better understand their unique roles during development, immune responses, disease pathogenesis, and maintaining homeostasis.

Historically, subsets of macrophages have largely been classified as pro-inflammatory (M1) or anti-inflammatory (M2) on the basis of expressing various markers, secreting cytokines, and phagocytic and antigen-presenting functions. Results from more recent research has suggested that the polarization of macrophage phenotypes is more fluid and less dichotomous than originally thought, based on the ability of macrophages to change phenotypes in response to changes in the surrounding environment within the tissues and vasculature (16). Generally, tissue-resident macrophages appear more M2-like with functions supporting an anti-inflammatory environment such as phagocytosing debris and promoting healing and growth. These tissue-resident macrophages are considered longer-lived as illustrated in the lung with alveolar macrophages (17). Macrophages recruited from circulating monocytes, in contrast, largely constitute the interstitial macrophages (IM) in the lung and subsets of perivascular macrophages in the brain and are thought to be shorter-lived. These have been classified as both M1-like and M2-like depending on immune homeostasis, infection events, and expression of MAC387 and CD163 differentiation markers, respectively (16). CD163, a hemoglobin/haptoglobin scavenger receptor associated with anti-inflammatory responses, is expressed on subsets of tissue macrophages as well as on monocyte populations, which are thought to derive from CD14+CD16− monocytes (18). Soluble CD163 shed from monocyte populations exerts anti-inflammatory functions, and studies showed that increased levels are associated with infectious and autoimmune diseases [reviewed in Ref. (19)]. MAC387 recognizes the L1 or Calprotectin molecule, and has been used to identify recently infiltrated macrophages associated with acute inflammation (20, 21). During infections, particularly in the case of HIV/SIV disease progression, increased monocyte turnover occurs in association with an accumulation of these various short-lived macrophage subsets in the tissues. This macrophage deposition seems to primarily occur in classical sites of AIDS pathology, including the brain, heart, lung, and gut tissues (15, 22–24). One study proposes that during acute infection M1-like macrophages are recruited into tissues to help fight infection, while M2-like macrophages accumulate later during chronic disease to dampen inflammation and repair tissues. The accumulation of both macrophage subsets seems to promote cardiovascular disease (16). Thus, the characterization of monocyte and macrophage subsets in relation to their number, phenotype, and function becomes increasingly important for predicting disease outcomes and subsequently attempting to ameliorate or reverse pathogenesis.

## HIV Infection

Human immunodeficiency virus is the etiological agent of AIDS and, since the beginning of this pandemic in the early 1980s, has caused the loss of over 40 million lives (25). HIV infection in humans follows a distinct pattern (26) starting with an eclipse phase during the first 1–2 weeks of infection, which constitutes a time when the virus replicates and disseminates while the immune response is developing and not yet effective at clearing the virus. HIV preferentially targets T cells for infection, using the CD4 receptor in combination with the CCR5 chemokine receptor to gain entry. Effector CD4+ T cells express the highest level of CCR5 and are depleted drastically early after infection (27) in what is known as the acute phase. This particular phase follows 2–4 weeks post-infection (pi) and is evident by high viral RNA loads in the plasma, and a reduction in peripheral CD4+ T cells. During the acute phase, there is a stark increase in detectable immune responses, most notably with higher levels of virus-specific effector CD8+ T cells and antibodies. The end of the acute phase and transition to the chronic stages of infection is characterized by a marked reduction in plasma viral load (VL) and establishment of a viral “set point” that appears to result from incomplete control of viral replication by adaptive immune responses with concurrent loss of target CD4+ T cells available for new infection. The chronic phase of HIV infection is accompanied by clinical latency that may last from 1 to 20 years in untreated individuals. Eventually CD4+ T cells decline to levels below 200 cells/μl in blood and infected individuals become susceptible to opportunistic infections such as *Pneumocystis jiroveci* (earlier named *P. carinii*), cytomegalovirus, *Cryptosporidium* spp., and *Mycobacterium tuberculosis*, and cancers such as Kaposi’s sarcoma [reviewed in Ref. (28, 29)]. These late-stage infections and cancers associated with a loss of CD4+ T cells define the onset of AIDS that ultimately leads to death (30). With the development and application of combination ART (cART), survival in HIV-infected adults has been extended by suppression of virus to undetectable levels and restoration of CD4+ T cell numbers to normal levels, thus preventing onset of AIDS (31). Instead, longer-surviving HIV-infected individuals administered cART may experience inflammation-associated chronic diseases referred to as HIV-associated non-AIDS (HANA) conditions (32).

Despite the large success of ART treatment for lifespan and clinical symptoms, it does not completely eliminate the virus, which persists in reservoirs. In general terms, a viral reservoir is defined as a virus-infected cell that persists despite treatment. A reservoir can be described as active or latent. Active reservoirs continue to produce virus, such as HIV or SIV, and in this case the virus actively produces virons using the cells machinery for transcription and translation. This could result from an infected cell being refractory to ART treatment because the drugs may not penetrate the cell membrane or intracellular drugs may be ineffective at inhibiting the reverse transcriptase or integrase enzymes. In such cases, viral RNA or protein should be detected if expressed above threshold limits of the assay used. Alternatively latent virus reservoirs develop after insertion of viral DNA into a host cell genome. Here, virus is not actively being transcribed and effectively remains dormant. In this case, viral DNA should be detectable with sensitive assays. Cellular activation and some chemical compounds (latency reversal agents) can stimulate the active transcription and translation of the dormant virus, wherby viral RNA and protein will then be produced. Resting CD4+ T cells are thought to serve as latent reservoirs of HIV and SIV during ART treatment, and some latency reversal agents have produced successful reactivation of those reservoirs in attempt to eliminate the infection with continued long-term ART treatment. This is a fascinating and growing area of study that we will not review here but is described in depth in other reviews (33, 34).

Interestingly, in human and macaques, macrophages and dendritic cells (DC) are also capable of expressing the CD4 and CCR5 receptors required for HIV/SIV infection (35–39) and earlier work shows that macrophages can be targeted by HIV (40, 41) and SIV *in vivo* (42). Initially, virus tropism for the T cell and macrophage CCR5 receptor was used to delineate specific strains of these lentiviruses (43). It was largely accepted that while macrophages were capable of being infected by HIV, they were second to infection of CD4+ T cells, and possibly only targeted after significant loss of CD4+ T cells in the host (44). This evoked some persisting controversies regarding macrophage infection by HIV/SIV. *In vitro* studies showed that macrophages can be infected productively (45–47), and several strains of virus were considered macrophage-tropic yet *in vivo*, these same strains predominantly infected CD4+ T cells. *In vivo* data revealed that alveolar macrophages collected from bronchoalveolar lavage (BAL) in ART-treated human patients were positive for HIV DNA, indicating they are likely an important macrophage reservoir (48). Contradicting reports have indicated that while HIV DNA could be found in ART-treated human alveolar macrophages, subsequent outgrowth assays failed to detect any replication-competent virus, attributing positive DNA results to macrophage engulfment of infected CD4+ T cells (42). Work performed with rapidly progressing adult macaques (SAIDS progression occurring in months compared to years) reported detectable SIV RNA in macrophages of the lung and lymph nodes (LNs) (49). For example, Avalos and colleagues recently found that in virus-suppressed ART-treated macaques, macrophages isolated from the brain harbored replication-competent virus. These macrophages were designated as latently infected given that viral DNA was detectable, while *in situ* hybridization and qPCR of brain tissue showed no detectable viral RNA. These infected macrophages were found after 2 years of ART treatment, supporting the notion that they constituted a long-lived macrophage reservoir (50). Alternatively, Dinapoli and others detected SIV DNA and replication-competent virus in lymphoid macrophages isolated from ART-naïve macaques, but they were unable to detect viral outgrowth from similar macrophage populations in ART-treated animals. They suggested that infected macrophages were short-lived and were lost after longer treatment periods (42). In the humanized mouse model, results showed productive infection of macrophages as well as viral rebound after successful ART treatment in myeloid-only mice (MoM), which lacked human T cells (51). The viral rebound in the MoM study supports infection of long-lived tissue macrophage populations because short-lived macrophages have a half-life of 1 day and viral rebound occurred 7 weeks after withdrawal of ART (52). Additional studies in macaques have also reported infection of long-lived tissue macrophages and together these findings support the theory that macrophages can contribute to the viral reservoir (53, 54).

There is increasing attention on the characterization of macrophage phenotypes and functions during active HIV/SIV infections to better understand their roles in immune responses and tissue pathogenesis. For example, Burdo and colleagues found that an increase of MAC387+ macrophages in the central nervous system (CNS) was associated with more damage to the dorsal root ganglia, which is related to neurological complications in HIV infection (55). They also found that while MAC387+ macrophages were within brain lesions during acute SIV encephalitis (SIVE), higher numbers of CD163+ macrophages were associated with severe SIV encephalitic lesions during chronic infection. Additionally, an increase in CD163+CD16+ monocytes was found in HIV-infected patients with detectable viral loads despite ART treatment. The numbers of these cells were inversely correlated with numbers of CD4+ T cells among patients with <450 CD4 T cells/μl, suggesting that the CD163+CD16+ monocytes were contributing to viral replication (18). Another study reported increasing CD163 expression on monocytes in HIV-infected, ART-treated patients that inversely correlated with CD4 T cell number, however, this did not correlate with viral load (VL) and the use of protease inhibitors during treatment resulted in reduced shedding of CD163 on monocyte populations (56). In addition, increased monocyte turnover in the periphery was associated with increased trafficking of CD16+ macrophages into the gut tissue of chronic SIV-infected, progressing rhesus macaques (24). These gut macrophages were skewed functionally for anti-inflammatory responses and exhibited impaired phagocytic ability, which in turn was associated with increased disease of the gut. In *ex vivo* studies, monocytes expressing CD16 were preferentially infected by HIV over CD16-negative populations, and HIV DNA was detected in this cell subset sampled from ART-treated patients, also suggesting that these monocytes may serve as a viral reservoir (57).

Importantly, in contrast to T cells, macrophages and DC are described as more resistant to the cytopathic effects of the virus infection (47, 58, 59) and are not cleared by antigen-specific cytotoxic CD8+ T cells (60). Thus, in theory, infection of long-lived macrophages would allow the virus to rapidly evade CD8+ T cell immunity and replicate to produce “escape” mutations that then preferentially infect CD4+ T cells. As CD4+ T cells are depleted, B cell responses for antibody production may become less effective. These events would then promote widespread activation and infection of macrophages that survive and enable continued virus replication and release for long periods of time. Macrophages are clearly capable of contributing to disease progression, treatment failure, and viral rebound. Thus, our studies are currently devoted to depleting macrophages during various stages of SIV infection to develop a clearer understanding about their role in disease, with and without concomitant T-cell depletion.

Despite recent advances in antiretroviral therapies for treatment of HIV, the question of an HIV-infected macrophage reservoir has become significant as efforts are aimed at developing a cure. It has been shown that nucleoside reverse transcriptase inhibitors and/or protease inhibitors were less effective at targeting infected macrophages while being more effective against virus-infected T lymphocytes in SIV-infected rhesus macaques (61, 62). Also, macrophages may be productively infected by mechanisms different from CD4+ T cells such as by phagocytosis of infected cells (63) or by direct spread of infection between monocyte-derived macrophages using nanotubes (64). Macrophages can also harbor cell-free virus and spread infection to lymphocytes *in trans* using lectin receptors (e.g., CD169) (65). In addition, the mean half-life of HIV DNA was found to be longer in monocyte populations compared to CD4+ T cells (66). Together, these findings highlight the need to characterize macrophages in relation to disease pathogenesis and for developing effective treatments to eliminate this population as a viral reservoir.

## Rhesus Macaque Model for HIV

Non-human primates experimentally infected with SIV, primarily Indian and Chinese rhesus macaques, have been used to study the pathogenesis, immunology, and therapeutic interventions. They display a similar course of infection and disease progression pattern as seen in persons infected with HIV but at a faster rate. Rhesus macaques inoculated with SIV typically exhibit a high initial viral load, significant depletion of CCR5+CD4+T cells in the gut, and rapid seeding of virus in the tissues followed by establishment of the viral set point and gradual loss of CD4+ T cells in the periphery. After a few years, SIV-infected macaques develop SAIDS and succumb to opportunistic infections and wasting (67, 68).

Compared to HIV in humans, SIV in rhesus macaques uses the same cellular targets and anatomical sites, displays similar persistence, latency and viral loads, and is similarly responsive, though to a lesser degree, to the cART regimen used in human patients (69). The SIV-infected macaque model has enabled more detailed studies about cART therapy, viral seeding of reservoir cells, immune cell depletion effects, administration or generation of neutralizing antibodies, viral rebound, vaccine therapies and latency reversal agents [reviewed in Ref. (69)], much of which could not be examined to the same degree in human patients. Most importantly, animal studies have assisted in the development of effective drug treatments, advanced to producing undetectable viral loads and prolonged maintenance of normal levels of CD4+ T cells to thereby extend a long asymptomatic lifespan to HIV-infected individuals. However, even with strict adherence to cART therapy, drug treatment does not completely eliminate latent virus or virus production from long-lived host cells, and disruption of treatment consistently results in HIV viral rebound from a cellular reservoir [reviewed in Ref. (70)]. Viral seeding studies in macaques showed that even with ART initiation 3 days pi, prior to detectable VL or antigen-specific immune responses, viral rebound occurred when treatment was stopped. While this observed viral rebound was delayed, the post-rebound set points were similar to those observed in animals with ART initiation 2 weeks pi (71). Notably, studies performed in NHPs have found that viral reservoir seeding can be accomplished within 24 h of infection, and ART initiated as soon as 4 h pi, while beneficial in reduction of viral load, dissemination and reservoir seeding, was still not curative (72, 73). Taken together these studies highlight the difficulty and necessity of defining the viral reservoir and for eliminating long-lived or proliferating reservoirs to achieve cure. Much work has been devoted to investigating the role of memory CD4+T cell populations as a reservoir but other host cells, particularly long-lived macrophages, require attention as well due to their being refractory to ART and their potential to harbor virus for years.

## SIV Infection and Disease Progression in Adult Rhesus Macaques

**Figure 2:**
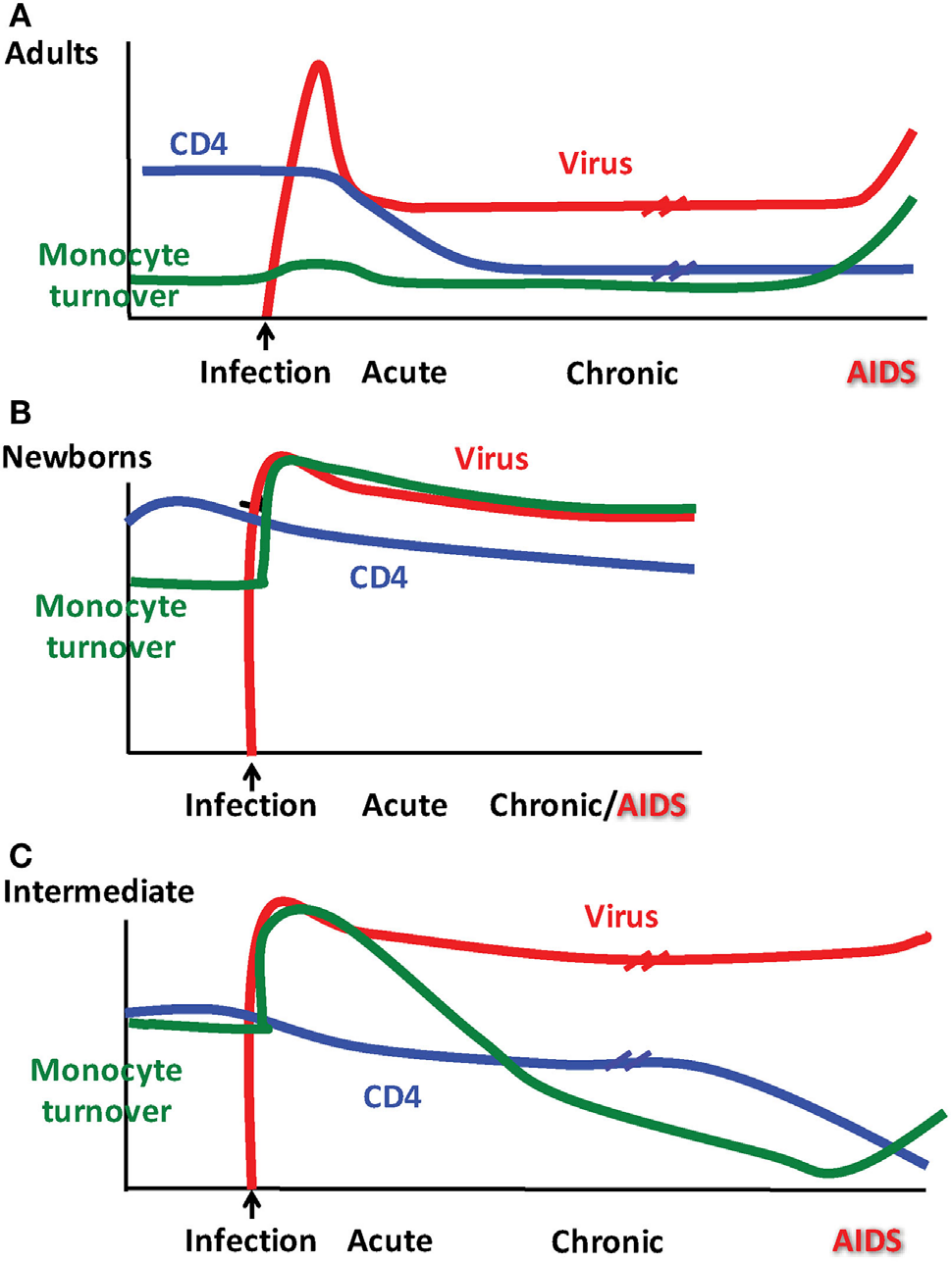
Patterns observed for monocyte turnover, CD4 T cell counts and simian immunodeficiency virus (SIV) infection differ depending on age at infection. Results from Kuroda and colleagues showing monocyte turnover determined by quantification of bromodeoxyuridine (BrdU) incorporation by flow cytometry in monocytes at 24 h post-BrdU intravenous injection, CD4 T cell count obtained by peripheral blood cellular immunophenotyping *via* flow cytometry in conjunction with complete blood count (CBC), and SIV RNA plasma viremia as determined by quantitative PCR. Distinct patterns emerged for clinical progression of SIV in rhesus macaques illustrated in **(A)** adults, **(B)** newborns, and **(C)** as what we refer to as an “intermediate” phenotype in older infants infected at ~3–4 months of age.

The typical course of SIV infection in rhesus macaques varies with age of the host as depicted in Figure 2. Early after infection, adult macaques produce a high initial viral load at ~10^8^ RNA copies per ml followed by a set point at ~10^5^ viral copies per ml that corresponds to a loss in CD4+ T cell numbers in the blood, all of which resemble HIV infection kinetics in adult human patients (74, 75). Disease progression and onset of AIDS/SAIDS is described typically using parameters such as CD4+ T cell count <200/μl with significant increase in viral load, and presentation with opportunistic infections, weight loss, anemia, pneumonia, fatigue, diarrhea, thymic atrophy, lymphoid atrophy, bone marrow hyperplasia, encephalitis, and colitis. Common reported opportunistic infections with SAIDS include cytomegalovirus, adenovirus, *Cryptosporidium, Pneumocystis, Mycobacterium, Shigella*, and *Campylobacter* (76, 77).

One of the many focal points in HIV/SIV research has been to characterize the physiological “flashpoint” and reliable prognostic markers that define onset of AIDS. Depletion of CD4+ T cells has been described as the primary cause for terminal progression to AIDS. Interestingly, however, CD4+ T cells can be depleted or remain at stable low levels for years before SAIDS onset occurs in rhesus macaques (78–80). In addition, CD4+ T-cell depletion or turnover has proven to not be the best predictive value for onset of terminal disease progression (80, 81). A recent working theory is that the depletion of CD4+ T cells occurs in three stages during the chronic stages of infection prior to AIDS onset such that initially effector memory (EM) T cells with high expression of CCR5 are depleted. This is followed by immune reconstitution of this population by CCR5-negative central memory (CM) cells and then ultimately leads to progressive loss of both effector and CM T cell pools over time with repeating cycles of reconstitution and depletion (27). It is thought that the loss of CM T cells leads to dysregulation of the immune network as a whole and immunodeficiency allowing for opportunistic infections that define the onset of AIDS (27).

During studies to further define biomarkers for the onset of SAIDS, we reported that increasing monocyte turnover with corresponding destruction of tissue macrophages was a significantly better predictor than declining CD4+ T cell counts or increasing plasma viral load (81), as diagrammed in Figure 2A. This suggested that in untreated adult rhesus macaques, macrophages play important roles in HIV/SIV pathogenesis and persistence. Additional studies show that they do so, in a tissue–specific manner as elaborated further in the next section.

## The Role of Macrophages in Adult SAIDS

Increases in peripheral CD16+ monocyte subsets have been associated with inflammatory disorders such as cardiac disease (82), lesions within the kidney vasculature during systemic lupus erythematosus (83), rheumatoid arthritis (84) and intestinal pathology (85). A subset of CD14+ macrophages has been linked to greater inflammatory damage in intestinal tissues, impacted by Crohn’s disease (86, 87). To also characterize the relationship between macrophages and SIV pathogenesis, we measured turnover rates of monocytes during steady-state homeostasis in uninfected macaques in comparison to SIV-infected rhesus macaques. Monocyte turnover was followed by measuring uptake of the thymidine analog, bromodeoxyuridine (BrdU), into dividing cells followed by immunofluorescent antibody staining and flow cytometry. The homeostatic baseline monocyte turnover in the blood of uninfected adult rhesus macaques was ~5% across several study groups, while the turnover of monocytes increased up to 50% prior to onset of terminal SAIDS (81). This agrees with data in a study where increased turnover of monocyte populations in the blood was predictive of more severe disease progression, in particular the development of SIVE (88). This was also related to an increase in macrophage populations deposited in the brain tissue, leading to localized pathology. Our study showed that an increase in monocyte turnover kinetics was associated with a loss of tissue macrophages by SIV infection in the LNs, and that this was not accompanied by changes in the absolute count of monocytes in peripheral blood (23, 54, 81). Interestingly, the shift in monocyte turnover was also not linked to numbers of CD4+ T cells or lymphocyte activation, similar to results reported by Burdo et al. (88), suggesting an independent mechanism of disease pathogenesis relating specifically to monocyte/macrophage infection (81). Further evaluations found that increased trafficking of monocytes from the bone marrow during SIV infection did occur with successive recruitment to the LN, CNS, and lung (23, 54, 88). In fact, increased rates of monocyte turnover and recruitment to become tissue macrophages were linked to rate of progression to SAIDS, severity of lung tissue pathology, and SIV encephalitis.

The lung, followed by the brain, appears to be among the organs most affected by HIV in the post-ART era (89, 90) with macrophages thought to play an important role in the chronic immune activation required for lung and brain lesions associated with chronic HIV/SIV infection. The accrual of CD163+ macrophages has also been observed in the hearts of rhesus macaques infected with SIV, which positively associated with cardiac disease (22). Given that respiratory infections such as *Pneumocystis* pneumonia and tuberculosis are among the most common AIDS-defining diseases and involve direct bacterial infections of macrophages, we focused further on characterizing macrophages of the lung. At least two different populations of macrophages were identified in the lung tissue of rhesus macaques; namely IM (23) and macrophages located in the alveolar spaces (AM) (91). BAL macrophages did not entirely represent macrophage-mediated innate immune responses of the lung, so we examined macrophages in whole lung tissues and observed that IM and alveolar macrophages (AM) exhibited different turnover kinetics and phenotypes in adult rhesus macaque lung tissue. IM were smaller, located exclusively in the interstitium, and phenotypically resembled blood monocytes. *In vivo* BrdU incorporation by dividing cells demonstrated higher turnover rates for blood monocytes and lung IM compared to AM during steady state (91). The relatively higher TUNEL-staining IM also suggested a continuous transition of blood monocytes replacing apoptotic IM in an effort to maintain homeostasis in the lung. These data led us to believe that IMs are a short-lived population of macrophages in the lung. AM on the other hand, were larger, expressed CD206 (not expressed on IM), and were located in the alveolar spaces. AM turnover was negligible during steady state indicating that these are longer-lived cells. In addition, *in vivo* BrdU labeling suggested that IM can differentiate into AM when AMs are depleted (91). These findings suggest that AM and IM possess different functions.

**Figure 3:**
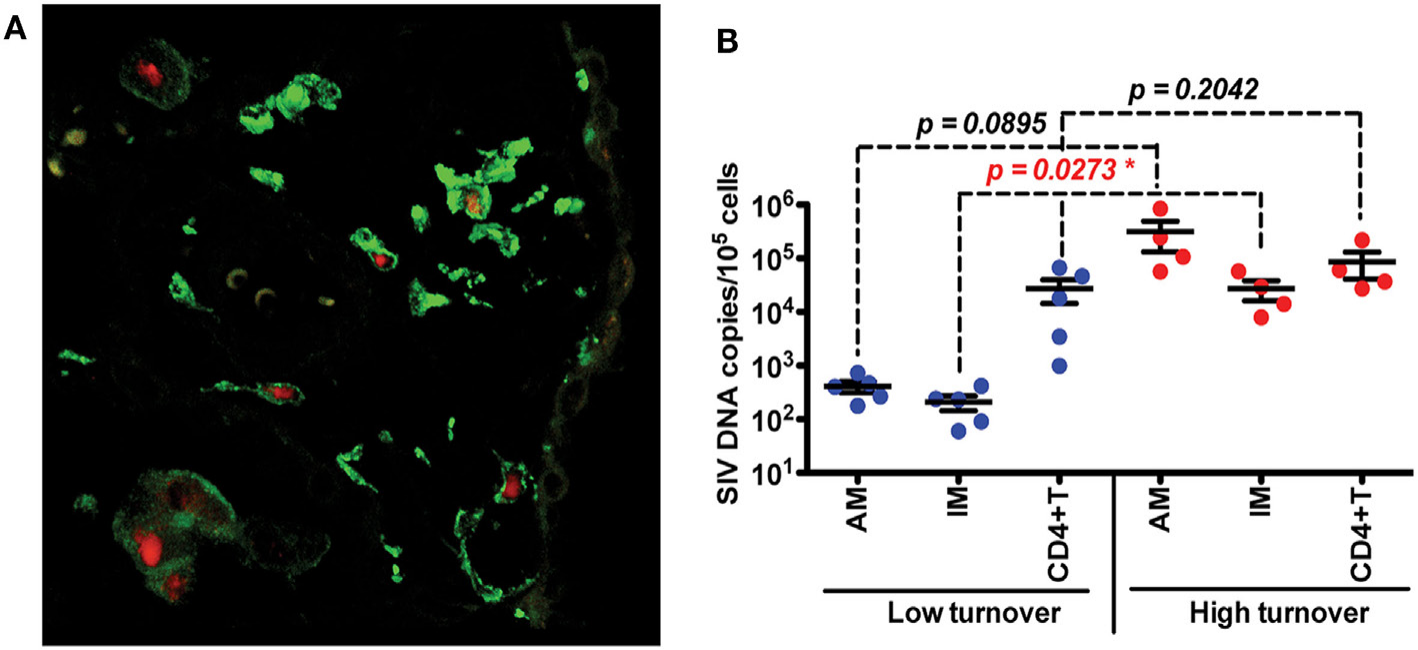
Infection and replication of simian immunodeficiency virus (SIV) in interstitial macrophages (IM) and AM contribute to the viral load in lung tissues and higher death rate of lung IM correlates with increased blood monocyte turnover rate in adult SIV-infected rhesus macaques. **(A)** Confocal microscopy was performed on lung tissues obtained from SIV-infected monkeys with higher (>30%) blood monocyte turnover levels (*n* = 6). Anti-CD163 (Green) antibody was used to identify macrophages, and SIV RNA (Red) was detected with anti-sense riboprobes. **(B)** IM, AM and CD4+ T cells were sorted *via* FACS from single-cell suspensions of lung tissues from SIV-infected monkeys with low (*n* = 5) and high (*n* = 4) blood monocyte turnover, and SIV DNA levels were quantitated and standardized against RNase P levels. Figure modified from Ref. (54). Copyright 2015. The American Association of Immunologists, Inc.

**Figure 4:**
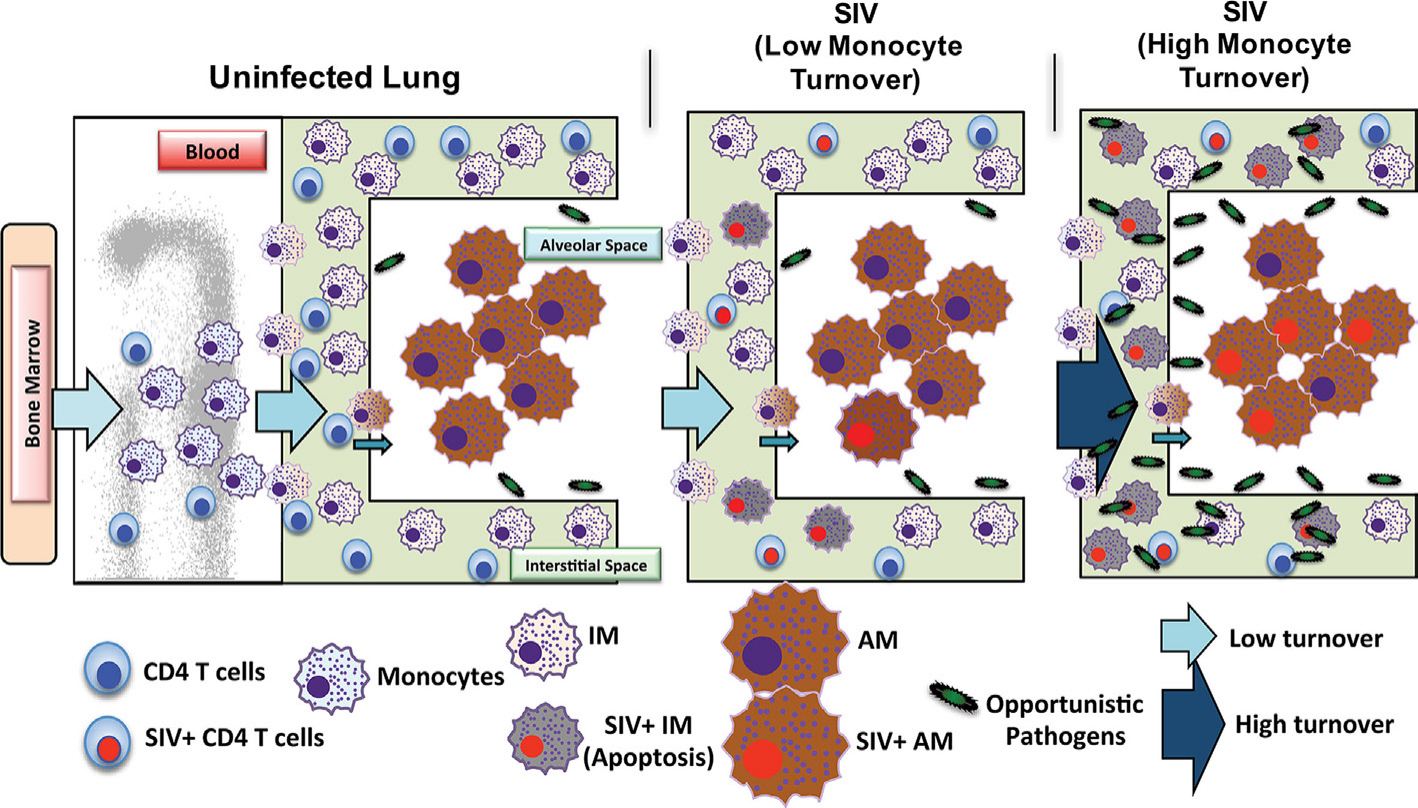
Proposed mechanism of simian immunodeficiency virus (SIV) reservoir establishment in the lung of SIV-infected macaques. The lung contains at least two types of macrophages: short-lived interstitial macrophages (IM) and longer-lived alveolar macrophages (AM). IM become massively infected with SIV and undergo a high rate of apoptosis that correlates with increased blood monocyte turnover in SIV-infected newborn macaques from the beginning of infection (Acute). This is in contrast to SIV-infected adults where massive infection of macrophages occurs later during disease progression. Conversely, SIV infection of the longer-lived AM does not lead to a high rate of apoptosis compared to that of IM. Elimination of SIV-infected longer-lived AM, and CD4+ T cells, is crucial for reducing SIV/HIV reservoirs and consequently reducing lung tissue damage.

Investigations of BAL collected from HIV-infected patients have produced some conflicting data and merited some interesting questions about whether AMS are productively infected and contribute to the viral reservoir. Results from several studies have found viral DNA and RNA in AMs (92–95) and some work has even defined a specific subset of HIV-specific CD8+ T cells whose function is thought to target HIV-infected alveolar macrophages (92, 96). There remains, however, a persisting argument that these results reflect macrophages engulfing infected CD4+T cells rather than true infection and replication within macrophages. In one study, viral DNA was found in AMS collected from a patient after 3 years of ART treatment, but the levels of rearranged TCR DNA supported their interpretation that viral DNA was present due to phagocytosis of infected CD4 T cells rather than macrophage infection (42). We thus investigated the effects of SIV infection on lung macrophage populations in ART-naïve macaques. Confocal imaging techniques were applied and lung macrophages were identified containing SIV RNA Figure 3A. This strongly supports the prospect that viral replication can occur in tissue macrophages. Furthermore, data showed that increasing blood monocyte turnover significantly correlated with turnover and apoptosis of IM but not with turnover of AM in SIV-infected macaques during terminal disease progression as shown in Figure 3B (54, 91). Interestingly, virus DNA copies increased in IM and AM after monocyte turnover increased in SIV-infected macaques but remained at the same levels in CD4+ T cells regardless of monocyte turnover (Figure 3B). The low turnover rate of AM despite their being infected with SIV strongly suggested that if infected with replication-competent virus, this macrophage subset could constitute an important long-lived virus reservoir (23, 91). Our interpretation is that the increased monocyte turnover was due to a compensatory mechanism to replace the short-lived macrophages (IM), which were destroyed by SIV in the tissues. This increase in turnover of both monocytes and IMs corresponds to greater infection of long-lived AMs, however mechanisms for this relationship are unclear. Based on these studies we proposed a working model as shown in Figure 4 that (a) There exist at least two types of macrophages in the lung including the shorter-lived IM exhibiting relatively higher turnover during homeostasis suggesting a critical role for daily protection, and longer-lived AM that exhibit lower turnover rate; (b) Increased SIV infection of both IM and AM correlates with AIDS disease progression; (c) SIV-induced IM apoptosis promotes further increases in IM (and monocyte) turnover rate in contrast to AM that become infected with SIV but exhibit far lower rates of apoptosis and turnover. This further suggested that similarly longer-lived macrophage populations in other tissues/organs also may become infected, and because they appear to be resistant to apoptosis, are likely also to support viral infection for extended periods of time to serve as a virus reservoir and source of chronic inflammation. Together, these studies illustrate the rationale for our proposed macrophage depletion strategies.

Simian immunodeficiency virus-infected macrophages have appeared to promote pathogenesis in various tissues resulting in cardiovascular, metabolic and neurological diseases. Research on the CNS has shown that HIV/SIV infection in the brain was associated with accumulation of perivascular macrophages and microglia associated with lesions and encephalitis (15, 97–99). Attention is also being given to adipose tissues, which are capable of influencing systemic immune responses and are thought to contribute to chronic inflammation seen in aging and with some infections (100–102). Adipose tissue macrophages were found to be associated with increased inflammation and lipodystrophies during HIV/SIV infection (103). Infection with SIV resulted in phenotypic and functional changes in resident cells of adipose tissue, with overall increased immune activation. SIV-infected macrophages and memory CD4+ T cells were observed in the stromal vascular fraction of the adipose tissue studied and have been proposed as potential reservoirs during ART treatment, particularly as adipose may be a site not fully permissive to ART drugs (104, 105). In addition, virus-infected macrophages identified in bone marrow also correlated with hematologic abnormalities, anemia, and bone marrow hyperplasia in SIV-infected macaques (106) and may migrate to the CNS causing HIV-1-associated dementia in humans (107). Other studies on the gut mucosa in humans showed that infected macrophages accumulating in the ileum exhibited increased inflammatory profiles, and thus likely contributed to intestinal dysregulation associated with AIDS onset (108). Furthermore, HIV/SIV infection produced changes in splenic architecture associated with significant shifts in macrophage populations expressing CD68, CD163, and Mac387 in this organ (109). A recent study in humanized mice determined that longer-lived splenic macrophages could harbor latent HIV during ART treatment (110), which would not only account for viral persistence, but also conceivably contribute to macrophage dysfunction and chronic immune activation. Thus, a better understanding about different outcomes of HIV/SIV infections in short- and long-lived macrophages will be critical to fully characterize mechanisms of HIV pathogenesis and establishment of virus reservoir to ultimately achieve a cure for AIDS. Although the molecular mechanisms are yet unclear, data from our rhesus macaque model demonstrated that SIV infection and reduced half-life of the short-lived macrophages appears to contribute to pathogenesis and AIDS disease progression. By contrast, long-lived macrophages that also are susceptible to SIV infection, do not exhibit a reduction in half-life because they survive infection. The long-lived macrophages could, therefore, be the perfect site of a long-term virus reservoir and a source of chronic inflammation observed in HIV-infected individuals.

## SIV Infection and Disease Progression in Pediatric Rhesus Macaques

HIV/SIV infections produce rapid rates of disease in infants compared to adults and infants also exhibit an immune system that is overwhelmingly biased toward immunosuppression or anti-inflammatory responses [reviewed in Ref. (111)]. Pediatric immunity is often referred to as “impaired” due to minimal or absent Th1 responses that have not yet fully developed. *In utero*, babies are protected in a semi-sterile environment and must remain immunologically tolerant to maternal antigens (112). After birth, neonates are bombarded with new foreign substances and organisms, many of which are considered harmless, or even beneficial. Prior to and during maturation of the immune system, neonates are partially protected by maternal immunity through transplacental transfer of antibodies and via breast feeding which allows for passive transfer of antibodies, lymphocytes, immune factors, as well as by seeding the neonate’s gut with commensal bacteria that ultimately help regulate responses to infections and avoid deleterious inflammatory responses [reviewed in Ref. (113)]. Tolerance to many benign antigens early in life would be beneficial for the infants survival, coupled with a gradual recognition and ability to immunologically discriminate self from non-self antigens. The transition from tolerance to expression of mature effector immune responses occurs over several years and occurs asynchronously between immune compartments, with full immune-competence achieved at adolescence (114). However, it is important to consider that the initial lack of inflammation in a newborn and the slow maturation with development could also favor an infection to spread freely and reproduce exponentially, highlighting the important balance between initial tolerance mechanisms and eventual maturation of a Th1-capable immune responses. This also highlights the influence of age at infection, which could dictate severity of disease, depending on the pathogen and the immune-competence of the child at time of infection. Studies using infant macaques have proven vital for examining immune ontogeny during fetal and postnatal development due to their physiological similarities to humans and because they present with similar infection and disease outcomes after SIV infection compared to pediatric HIV infections (115). Unanswered questions remain, however, regarding pediatric development of competent immune responses in the context of the infection and time course of disease.

**Figure 5:**
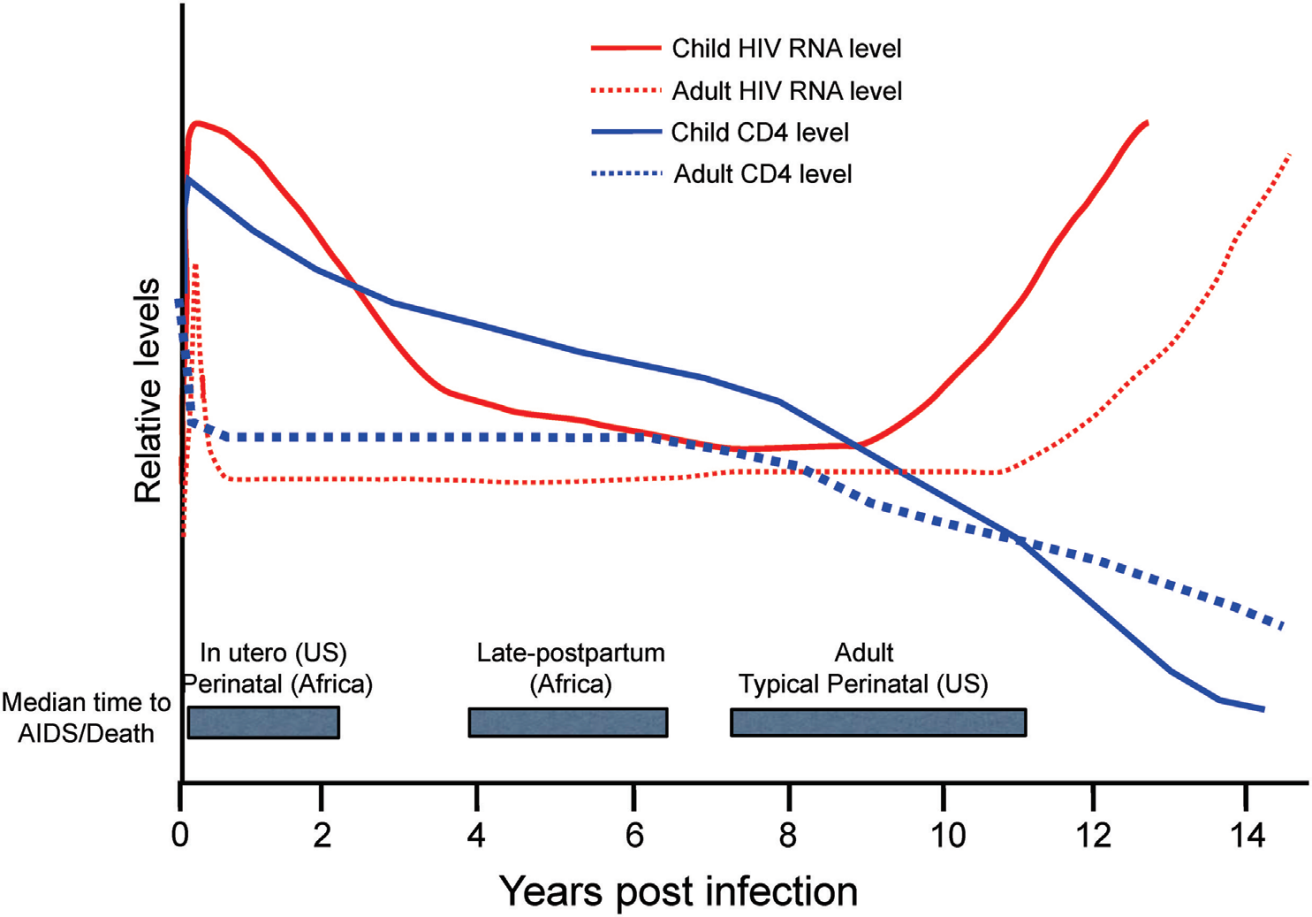
Relative levels of human immunodeficiency virus (HIV) RNA (red) and CD4 cells (blue) in adults (dotted lines) and children (solid lines) in the years following acquisition of HIV-1. Gray boxes highlight the median time to AIDS/Death for infants by region and route of infection in comparison to adults. US, United States. Figure from Ref. (114) © 2013 John Wiley & Sons A/S. Published by John Wiley & Sons Ltd. Immunological Reviews 254/2013.

The rapid disease and higher mortality rates, which occur in pediatric cases, suggest that HIV and SIV are able to exploit the immature immune system in infants. As shown in Figure 5, total CD4+ T cell numbers are significantly higher in infants than adults (116, 117). The primary target cells of the virus are the CCR5+ fraction of CD4+ T cells, but these cells are lacking in the blood and LNs of infants (118). In humans, a biphasic pattern of disease progression occurs in untreated, HIV-infected children, with half reaching terminal AIDS by the age of two (119, 120), while the other half present with slower onset of disease, and survival through adolescence (121). In both groups of HIV-infected children however, acute plasma viremia generally increases 10-fold above levels seen in adults, and RNA levels do not decline to reach a viral set point until ~5 years of age (122) (Figure 5). This suggests that CD4+ T-cell depletion and/or degree of immune activation do not consistently correlate with or promote disease progression to AIDS as has been shown with adult infections (123, 124). Based on our earlier results that higher monocyte turnover and macrophage destruction predicted onset of SAIDS in adult macaques (23, 54, 81), we hypothesized that similarly, the destruction and increased turnover of monocytes/macrophages, would be all the more illustrated in the setting of severe pediatric infection. Rhesus infants, similar to human children, have an increased number of CD4+ T cells systemically, compared to adults. Previous pediatric macaque work has shown that intestinal CCR5+ CD4+ T cells are preferentially infected by SIV and subsequently depleted (118, 125, 126). However, while these CCR5+ CD4+ T cell subsets have been observed in pediatric gut tissue, the vast majority of CD4+ T cells found in neonates, are known to express a naïve phenotype and are functionally biased for immune tolerance (125, 127–130).

These phenotypic and functional differences of CD4+ T cells in children, in addition to reported age-related declines of CD4+ T cells (131–133), further complicate interpretations about the impact of CD4+ T cell infection in pediatric cases that progress more rapidly to AIDS. Considering that neonates have fewer target CCR5+ T cells, and largely lack that hallmark CD4+ T cell depletion typical of adult infection (114, 117, 134), our attention turned toward studies on macrophages to better understand their contribution to exacerbated disease progression found in pediatric cases.

## Macrophages in Pediatric SAIDS

**Figure 6:**
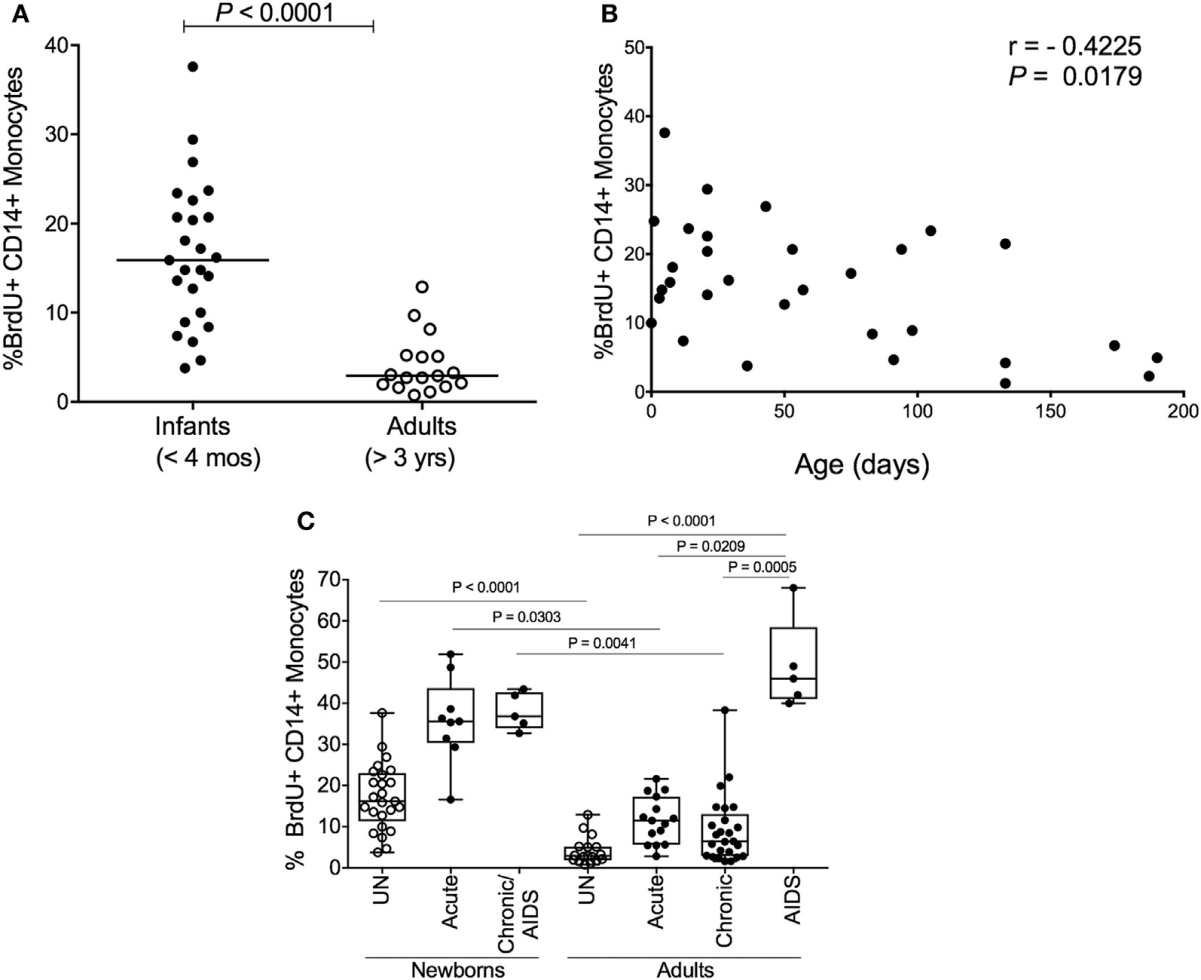
High monocyte turnover in uninfected infant rhesus macaques is further increased with simian immunodeficiency virus (SIV) infection. Bromodeoxyuridine (BrdU) incorporation was analyzed at 24 h by multicolor flow cytometry to determine the percent of BrdU+ monocytes (HLA−DR+ CD3− CD8− CD20− CD14+). Monocyte turnover rates were **(A)** compared between infant rhesus macaques and adult rhesus macaques and **(B)** examined in 20 uninfected macaques ranging from 3 to 190 days of age. **(C)** Statistical significance of comparisons between monocyte turnover rates of newborn and adult rhesus macaques before infections (UN = uninfected) and during acute, chronic and SAIDS stages of SIV-infected macaques were determined by Kruskal–Wallis test corrected for multiple comparisons using Dunn’s post-test; *P* < 0.05*, *P* < 0.01**, *P* < 0.001***. Figure modified from Ref. (135).

Since increasing monocyte turnover and tissue macrophage destruction by SIV predicted onset of AIDS in infected adult macaques, we sought to determine if monocytes/macrophages also influenced the more rapid and severe disease observed in SIV-infected infants. Currently, there is a distinct lack of published studies for role of macrophages in pediatric HIV/SIV infections. Baseline studies recently published demonstrated that naïve, uninfected neonate, and infant macaques aged 4 months or less inherently exhibited higher monocyte turnover rates (median of 15.9%) than observed in uninfected adult rhesus macaques (median of 2.9%) (135) (Figure 6A). By approximately 4–6 months of age, the rates of monocyte turnover declined to levels seen in adults (Figure 6B). This observation suggested that monocytes were undergoing maturation in function or physiology during the first few months of life in the rhesus macaques. This may have affected the findings that in animals infected with SIV at birth, monocyte turnover rates increased even further and remained high through their progression to SAIDS, without an intervening chronic stage as seen in adult infection (Figures 2A,B and 6C).

The physiologically higher monocyte turnover rates in the neonates appeared to reflect a developing, immature immune system that may pose a higher risk for macrophage infection by HIV/SIV. To examine this, we applied terminal deoxynucleotidyl transferase dUTP nick-end label (TUNEL) staining and observed apoptotic TUNEL-positive macrophages in LNs and intestine in SIV-infected neonates, which corresponded with increased trafficking of macrophages recently derived from circulating monocytes (BrdU+ CD163+). This was similar to observations in infected adult macaques progressing to SAIDS and suggested that the increased rate of monocyte turnover was necessary for reseeding tissue macrophages that were destroyed by the virus infection (135). Furthermore, immunofluorescence analysis of LN, gut and lung tissue demonstrated that a large proportion of the virus-producing cells were in fact macrophages (135). Taken together, the higher monocyte turnover in infants in concordance with damage to macrophages by SIV infection, may illuminate reasons for more rapid disease progression in SIV-infants as depicted in Figure 2B.

Despite similar levels of CD4+ T cells and plasma viremia between infected children, the age of the patient significantly influences the rate of terminal disease progression, with the youngest patients typically showing the highest mortality rates, as noted in Figure 5 with shorter median time to death found for *in utero* and perinatal cases of HIV infection. The rapid HIV disease progression in children begins to shift toward rates more similar to those seen in adult infections at about 5 years of age. This parallels the observations that in persons over 5 years old, predictive biomarkers of CD4+ T cell levels and plasma viremia measures also transition toward levels observed in adults (114) that likely signify maturation of the immune system. In pediatric studies, age-related differences were reported in innate immune responses (136, 137) as well as in lymphocyte populations comprising the blood and mucosal immune responses, including CD4+ T cells, CD8+ T cells, B cells, and NK cells [reviewed in Ref. (111, 138–140)]. Also, plasma molecules vary in concentration as a function of age (141–143).

**Figure 7:**
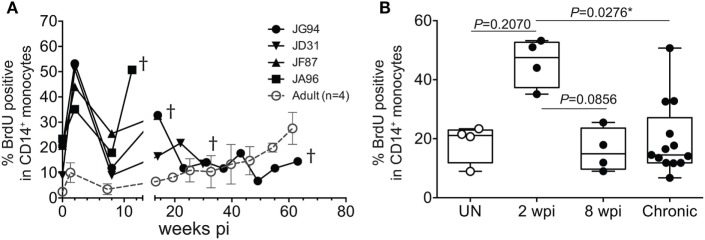
Longitudinal course of changes observed during progression to simian AIDS (SAIDS) in infant macaques infected with SIVmac251 at 3–4 months of age. **(A)** Bromodeoxyuridine (BrdU) incorporation was evaluated for monocyte turnover at 24 h. Blood monocyte turnover rates were measured every 4–6 weeks during the course of infection until progression to SAIDS or at indicated time-points (†). Mean monocyte turnover rates (i.e., %BrdU+ CD14+ monocytes) of the four infected adults are shown for comparison (dotted line). **(B)** Statistical analysis of monocyte turnover rates in infant macaques was performed by Kruskal–Wallis test corrected for multiple comparisons using Dunn’s post-test; *P* < 0.05*. UN; pre-infection, wpi; weeks post-infection. Chronic; all infant time-points after 8 wpi. Figure modified from Ref. (135).

To further assess the transition from pediatric to adult disease progression patterns, we experimentally infected slightly older pediatric animals aged 3–4 months of age when physiological monocyte turnover rate was transitioning to rates comparable to healthy adults. Of the four animals infected in this age group, two presented with viral load dynamics similar to animals infected as newborns, and they progressed rapidly with SAIDS diagnosis by 20 weeks pi. The SIV plasma viral loads in the remaining two infants were more comparable to levels seen in adults, and they exhibited a typical infection course, with acute to chronic phase similar to adult infections, rather than progressing directly to overt SAIDS as occurs in infected neonates (135). For these animals infected at 3–4 months of age, monocyte turnover increased dramatically during the acute phase, which was similar to that seen in the infected newborns (Figure 7A). However, instead of continuing to increase throughout infection, the monocyte turnover rates in the older infants declined to similar levels as adults (Figure 7B) at 8 weeks pi (albeit slightly higher) and were without changes in absolute number of monocytes in the periphery. Chronic phase was not only visible in the older infected infants but exhibited monocyte turnover rates more similar to adults before progressing to SAIDS. As clinical signs associated with SAIDS began to be observed, monocyte turnover increased again in the older infants, thus exhibiting what we consider an “intermediate” phenotype for monocyte turnover, plasma viral load, and risk for onset of terminal SAIDS progression in relation to divergent disease course progression rates seen in neonate and adult infections (135) (Figure 2C). This intermediate phenotype helps explain the biphasic disease patterns observed in HIV-infected children such that varying immune parameters at different stages of maturation at the time of infection appear to impact the rates of effective immune responses, disease progression, and survival outcomes.

These observations highlight the importance of using appropriate age-matched controls when studying pediatric infections and provide a foundation to continue work relating maturation of immune responses with changes in pathogenesis, immune activation, and dynamics in tissue macrophage populations in infants infected at various ages. To our knowledge little to no published work is available to compare and contrast our pediatric macrophage studies with, particularly in the case of HIV/SIV infection and AIDS progression. Additional considerations are required to better understand age-related immune responses and pathogenesis in patients undergoing ART that are routinely used in human and experimental macaque HIV/SIV infections. In general, ART is less effective at suppressing viral load in HIV-infected children compared to adults (144, 145). Typical outcome measures for treatment efficacy in children are viral RNA loads and CD4+ T cell counts, which have been highly variable compared to outcomes from similar drug protocols in adults, and especially when comparing different combination drug treatments (146). In addition, the definition of virologic suppression varies between studies that may show up to 30% failure rate after first line treatment (which does not include protease inhibitors) (146). The measures of percentage failures also are reported independent of death outcomes. Unlike in human studies, it is common to use more similar dosing regimens in rhesus macaque neonates and adults based on weight, which allows for more direct comparison between treatment efficacy and survival outcomes after experimental infections initiated at varying ages. Adults are often treated with a tri-regimen of emtricitabine, tenofovir and dolutegravir, and successful control of viral replication is routinely reported (69). Interestingly in pediatric macaques, drug therapies appear less effective than in adults, and the animals exhibit sustained high viral loads and undergo disease progression despite treatment (147). This is possibly associated with the accumulation of specific drug-resistant mutations in addition to a suppressed immune system. Our lab is currently evaluating the persistence of virus in tissue macrophages and T cells in pediatric animals treated with ART that integrate with monocyte/macrophage phenotype shifts to identify biomarkers of ART efficacy.

## Conclusion and Perspectives

Results from many studies have shown productive infection in macrophages, but their role as a latent reservoir is still highly controversial. In some studies, viral DNA is not detected in tissue macrophage populations after successful ART therapy. This could be due to the shorter half-lives of some macrophage subsets, small sample sizes, and/or threshold detection limitations in assays employed for detecting virus. It is often difficult to identify infected macrophages, especially in tissues that test negative by PCR or *in situ* hybridization, but viral outgrowth assays have exposed tissue macrophages as capable, latently infected reservoirs. Assay limitations in addition to limitations of HIV research in human tissues, encourages a continued debate. Our studies in rhesus macaques, however, show that short-lived macrophages contribute to active infection and then die, thereby inducing increases in monocyte turnover, which serves as a physiological biomarker to predict onset of SAIDS. Our correlate hypothesis then follows that long-lived macrophages become infected, do not die, and instead serve as a reservoir, refractory to ART. Susceptibility of the macrophage populations to virus infection is likely to be different between individuals, and is probably influenced by time of ART initiation, magnitude of VLs at ART initiation, viral strain, immune status, and age at infection. These variables could result in the debated inconsistencies found in the literature regarding the contribution of infected macrophages to viral reservoirs. It is important to note that while this review focused primarily on the role of monocyte/macrophages in HIV/SIV infection, other cells of the immune system may also be important influences on infection and pathology. Clearly, further investigations are necessary to identify and remove all viral reservoirs for development of therapeutic cures.

In summary, we have shown that increase in blood monocyte turnover, which results from death of short-lived macrophages in the tissue, predicts disease progression in SIV-infected rhesus macaques. Baseline monocyte turnover rates observed in uninfected neonates are higher than observed in uninfected adults. In animals infected with SIV at birth, monocyte turnover increased further and remained higher than in SIV-infected adults, and this was associated with a more rapid disease progression.

Our current studies are directed to study the connection between higher monocyte turnover and rapid progression in infants, as well as the contribution of SIV-infected macrophages as a viral reservoir. Work is in progress to evaluate the persistence of virus in tissue macrophages and T cells in pediatric animals treated with ART. We hypothesize that early and high infection of neonatal macrophages results in establishment of viral reservoir in long-lived subsets of macrophages, contributing to uncontrolled viral load despite ART and perhaps explaining failures of first line treatment in children.

Together, we review some important aspects of macrophages in SIV infection and progression, and highlight the need to further investigate their role in pediatric cases.

## Notes

Data presented from our own studies was carried out in accordance with the standards of the National Institutes of Health (NIH), Guide for the Care and Use of Laboratory Animals. The Institutional Animal Care and Use Committee of Tulane University approved protocols used. Permission has been obtained for use of copyrighted material from other sources.

## Author Contributions

All authors participated in the concept, preparation, and editing of the manuscript. KM wrote the manuscript and developed the figures.

## Conflict of Interest Statement

The authors declare that the research was conducted in the absence of any commercial or financial relationships that could be construed as a potential conflict of interest.
